# Amyloid‐beta (Aβ)‐targeting monoclonal antibody trials in early Alzheimer's disease—Clinical outcome with gantenerumab

**DOI:** 10.1002/ctm2.1559

**Published:** 2024-01-27

**Authors:** Bin Xiao, Eng‐King Tan

**Affiliations:** ^1^ Department of Neurology National Neuroscience Institute Singapore Singapore; ^2^ Neuroscience Academic Clinical Program, Duke‐NUS Medical School Singapore Singapore; ^3^ Neuroscience and Behavioural Disorders Program, Duke‐NUS Medical School Singapore Singapore

1

The field of therapeutics of Alzheimer's disease (AD) has recently witnessed major advancement, marked by the Food and Drug Administration approval of two amyloid‐beta (Aβ)‐targeting monoclonal antibodies.^1^, ^2^ Notably, the lecanemab study demonstrated that the compound was able to slow down cognitive impairment, accompanied by improvement of amyloid load on positron emission tomography (PET) scans.^2^

However, two recent phase 3 trials (985 and 980 participants each) involving participants 50–90 years of age with mild cognitive impairment or mild dementia due to AD reported that gantenerumab, a monoclonal antibody (similar to lecanemab) failed to show clinical benefits in the primary outcome measured by the Clinical Dementia Rating—Sum of Boxes (CDR‐SB) scale, a widely used cognition scale ranging from 0 to 18. The mean decline from baseline to week 116 has an insignificant difference of –.31 points and –.19 points between the gantenerumab and placebo groups.^3^

The trial findings may not be entirely unexpected. Compelling evidence suggests the involvement of Aβ in the pathogenesis of AD.^4^ The Aβ antibodies have been shown to remove the amyloid plaque, suggesting excellent target engagement in the brain. However, pathogenic effects of Aβ may occur before the formation of plaques. The reduction of amyloid plaque shown by brain PET only confirms the removal of extracellular Aβ but not those inside the neurons where the detrimental Aβ is generated and probably already have done the damage. The extent of the ability of the Aβ antibody to cross the neuronal membrane and establish target engagement inside neurons may vary or inconsistent, considering the antibodies were not specifically engineered to penetrate cell membranes.

Another possible reason could be that the drug specifically targeting Aβ may be insufficient for clinically relevant improvement in AD. Despite the unequivocal role of Aβ in AD pathogenesis, it is unclear if Aβ functions as the predominant driving force in AD pathology, and has a greater effect compared to the contributions of pathogenic tau or neuroimmune responses. The pathophysiologic heterogeneity and involvement of other microenvironmental and individual variability in responding to the amyloid load, may explain the differences in the trial results.^5^ In the clinical trial of donanemab, significant clinical benefits were observed in the participants in the low/medium tau group presenting stable scores of the CDR‐SB at 1 year, but this was not noticed in the high tau group.^6^ Hence, clinical and genetic heterogeneity in the AD population, coupled with variable intrinsic rate of AD decline could account for the negative findings in the gantenerumab clinical trials, which may represent a potential type II error.

One concern is the adverse event‐related discontinuation (9.1% in gantenerumab group and 1.8% in control group), which is mainly due to amyloid‐related imaging abnormalities (ARIA), especially ARIA with hemosiderosis (ARIA‐H), seen on brain magnetic resonance imaging. Although the trial reported only nearly twofold increase in ARIA‐H in gantenerumab group, the disproportional discontinuation rate indicates an association of gantenerumab with more severe ARIA‐H. The safety concerning over ARIA‐H has persisted in the clinical trials of monoclonal antibodies of Aβ since the report of the case of lethal multiple cerebral hemorrhages in a patient receiving lecanemab.^7^ We need more clinical data and focused trials in better stratified patients to evaluate the risk/benefit ratio before recommending monoclonal antibodies of Aβ to potential suitable patients. Despite the disappointing outcome of the two gantenerumab trials, it has helped highlight some of the pathophysiologic gaps and also lay out the challenges that need to be addressed in future immunotherapy trials (Figure 1).

**FIGURE 1:**
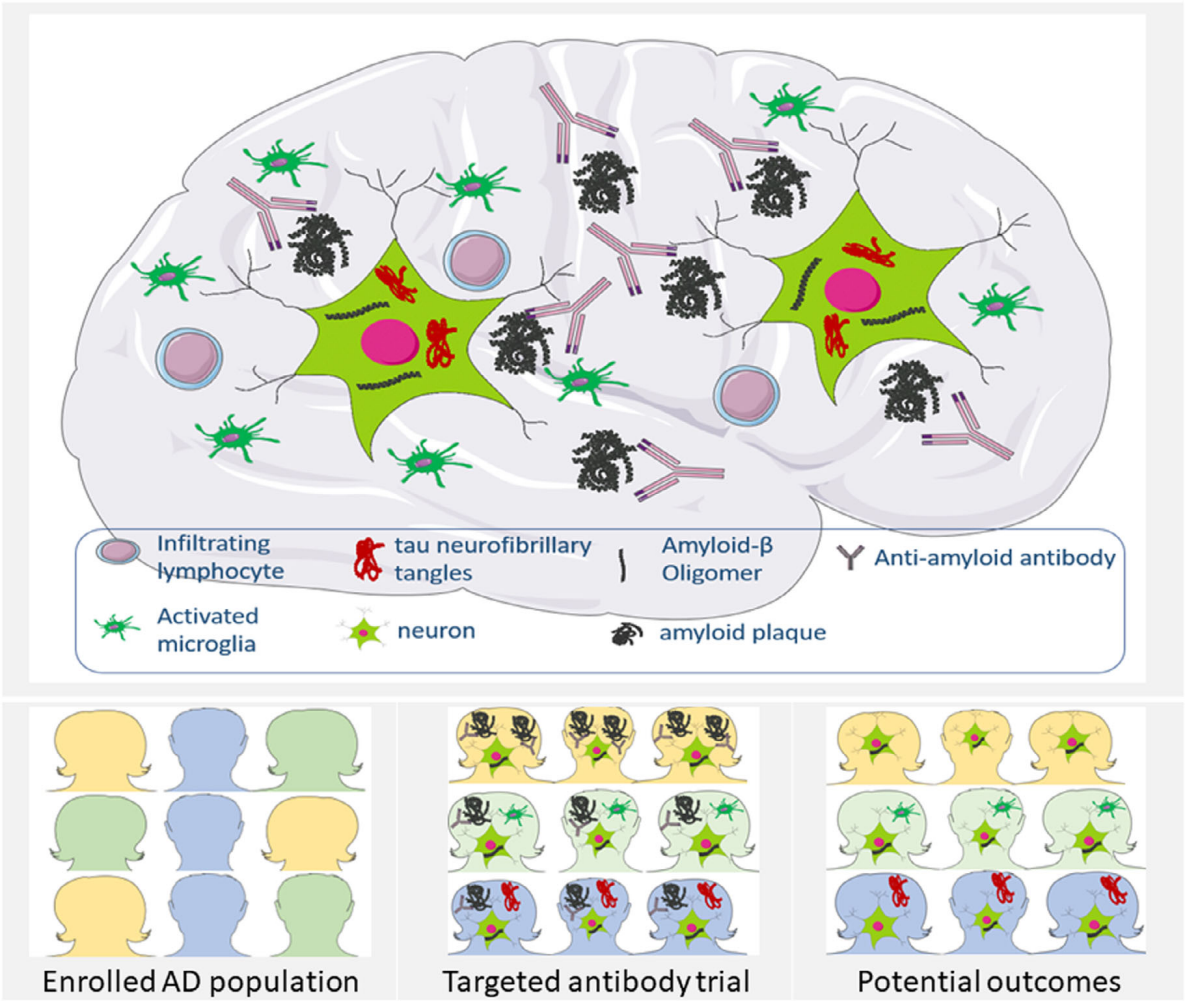
Potential implications of Aβ‐targeting monoclonal antibody in Alzheimer's disease (AD). The heterogeneity of AD is primarily attributed to the complicated underlying mechanisms, involving amyloid, tau, immune responses and other pathogenic factors. The clinical trials of monoclonal Aβ antibody have achieved success of removing amyloid plaque. But the limited clinical benefits may result from the selective impact of the antibody on Aβ while neglecting other causative factors. In addition, the target engagement of the antibody inside the neurons remains uncertain. Inclusion of pathologically relevant patients in the targeted therapy trials may contribute to the development of such therapeutics.

What can we learn from these Aβ‐targeting monoclonal antibody trials showing discrepant outcomes? Future strategies may benefit from a tailored approach involving drugs that act on the personalised profile of molecular pathways implicated in AD individuals, as identified and stratified by a panel of biomarkers.

## AUTHOR CONTRIBUTIONS

Both authors conceived and wrote the commentary, as well ascreated the figure.

## CONFLICT OF INTEREST STATEMENT

The authors declare they have no conflicts of interest.

## ETHICS STATEMENT

Not Applicable.
